# Physical and cognitive function to explain the quality of life among older adults with cognitive impairment: exploring cognitive function as a mediator

**DOI:** 10.1186/s40359-023-01087-5

**Published:** 2023-02-22

**Authors:** Rhayun Song, Xing Fan, Jisu Seo

**Affiliations:** College of Nursing, Chungnam National University, Munhwa-ro 266, Jung-gu, Daejeon, Korea

**Keywords:** Physical function, Cognitive function, Quality of life, Mediation, MCI

## Abstract

**Background:**

Physical and cognitive function are both indicators of aging, characterized by a loss of adaptive response to life challenges and functional limitations, subsequently affecting their quality of life. This study aimed to identify the direct effect of physical function and the indirect effect of cognitive function on the quality of life in older adults with mild cognitive impairment.

**Methods:**

The study participants were 79 older adults recruited from community centers in four urban districts of Korea. All participants completed a self-reported questionnaire for demographic characteristics and outcome variables. Outcome measures included physical function (grip strength, balance, and mobility), cognitive function, and mental and physical components of quality of life (QOL). Statistical analyses were conducted using hierarchical multiple linear regression and the PROCESS macro for mediation analysis.

**Results:**

The mean age of participants was 77.46 years old with an elementary or lower education level (53.2%). The mean score of cognitive function was 16.39 (SD = 6.5). Physical function (grip strength, balance, mobility) and cognitive function explained 25% of the variance in physical (p = 0.004) and 29% in mental (p < 0.001) components of QOL after controlling for age, sex, and education level. Mobility was associated with both physical (β=-0.27, p = 0.024) and mental (β=-0.36, p = 0.002) components of QOL. The cognitive function partially mediated the relationship between balance and the physical component of QOL; the proportion of mediation was 55%.

**Conclusion:**

In conclusion, physical and cognitive function were significant predictors of QOL in older adults with cognitive impairment. Specifically, balance has significant indirect effects on the physical component of QOL through cognitive function as a mediator. Health-promoting strategies could be more effective when focusing on the direct effect of physical function as well as the mediating effect of cognitive function to promote the quality of life in this population.

## Background

According to unprecedented aging growth, the number of older adults has been growing in the last few decades [1]. The impact of an aging population on healthcare systems is potentially overwhelming, particularly for age-related disorders such as dementia [1]. Mild cognitive impairment (MCI) is defined as the transition between normal cognitive aging and early dementia [2]. One of the key differences between people with MCI and those with dementia is how independent they are in their daily functioning [3]. The annual conversion rates from mild cognitive impairment to dementia are estimated from 10 to 15%, higher than the yearly incidence rate of dementia in the overall elderly population, calculated from 1 to 3% [4]. But there are no definite pharmacological treatments to prevent or delay cognitive decline [5]; it is necessary to develop effective interventions to support people with cognitive impairment to improve their quality of life.

Quality of life (QOL) is a crucial concept for understanding the impact of cognitive decline on those affected [6]. QOL is a subjective and multidimensional concept that consists of physical and mental well-being [7]. Over the lifespan, different aspects, including physical and mental well-being, become relevant for QOL [6]. A systematic review of 19 studies addressing QOL in older adults with dementia reported that functional impairment and depression were the significant influencing factors of QOL [8], supporting that physical health and mental health affect QOL in older adults with cognitive decline.

MCI, also associated with loss of daily functional living, was a substantial determinant in QOL [9]. Loss of function with aging has been linked not only to a reduction in physical function, such as difficulties in daily activities and decreased balance and grip strength [10], but also to cognitive functions, such as delayed processing speed, memory, language, and executive functions [11]. Stroke patients with cognitive impairment also had the worse balance of physical function since they needed a longer time to turn around or sit down [12]. On the other hand, physical function affects brain plasticity, influencing both cognitive function and quality of life [13], and deficits in physical function have also been associated with an increased risk of falls, diminished ability to live independently, and poorer health [14]. Significant positive correlations of physical and cognitive function across previous studies may strongly correlate with QOL, as it focuses on an individual’s activities and social participation, especially regarding participation in the general daily task as well as community and social interactions [15]. While much evidence shows that physical and cognitive function is associated with each other to explain the quality of life, the underlying mechanisms of their relationship and how they work together to induce beneficial effects on quality of life still need to be clarified.

Therefore, it has been postulated that improving physical function has beneficial effects on quality of life directly, while cognitive function may play a role as an intervening variable [16]. Several studies have found positive associations between physical and cognitive function in the elderly [17]. While aerobic exercise plus resistance training improved physical function and balance, leading to improved quality of life [18], 6-month aerobic exercise also improved cognitive function in older adults with MCI [19]. Greater levels of physical function (mobility, flexibility, and grip strength) were associated with better cognitive and executive function performance and improved quality of life in older adults [20]. Therefore, the association of physical and cognitive function should be considered when designing lifestyle interventions to improve QOL in this population.

With increased awareness of the efficacy of preventive and complementary intervention strategies for older adults with declining cognitive function, improved physical function may have a positive impact on the QOL through cognitive function as a mediator [21]. Despite evidence supporting the cognitive benefit of physical functioning, studies examining the mediating mechanisms underlying the physical-cognitive functioning relationship on QOL are still lacking. This study aims to explore the underlying mechanism of physical and cognitive function to explain QOL among older adults with cognitive impairment while focusing on cognitive function as a mediator.

Specific research questions were:

1. Do physical and cognitive functions predict physical and mental components of QOL in older adults with cognitive impairment after controlling for demographic characteristics?
2. Do cognitive function mediate between the indicators of physical function and QOL in older adults with cognitive impairment?

## Methods

### Study design

The secondary data analysis was conducted with the survey data from a larger intervention study [22], where data were collected from community-dwelling older adults with cognitive impairment, aged 75 years or above through a face-to-face, interviewer-administered survey. The original intervention study was approved by the Institutional Review Board of Chungnam National University Hospital (reference number: CNUH 2018-07-070), where the researchers were affiliated.

### Participants

From March to September 2020, all participants were recruited from community centers in four urban districts of South Korea. The inclusion criteria were (1) aged 75 years or older; (2) living in urban or rural communities; (3) scores on a test of K-MoCA less than 22; (4) being able to understand and communicate during the interview. Those currently hospitalized or living in long-term care facilities were excluded from this study. Eligible participants signed an informed consent form and participated in an interview to fill out the structured questionnaire in a meeting room of the centers. The participants of our study, including those with no education, were able to sign their names on the consent form after an interviewer explained the purpose of the study. A total of 135 candidates were screened for eligibility and 79 participants were enrolled in the study and completed the measures.

### Measures

#### Physical function

Measurements of grip strength, Time Up and Go (TUG) for mobility, and One Leg Standing (OLS) for balance were used in the study to assess the physical function of older adults [23].

*Grip Strength* was measured with a dynamometer (GRIP-D; Takei Ltd, Niigata, Japan). The participant was asked to squeeze the dynamometer with maximal effort, twice with each hand. An average score was recorded from both hands [24].

*Mobility* was measured by the *TUG test* with a stopwatch, timed from the point when the participants stood up from the chair to walk 3 m distance, turned around, and walked back to the chair till they sat down. The recorded time on the stopwatch in seconds is the value of TUG [25]. A less score of TUG represents better mobility.

*Balance* was measured by one leg standing with eyes open (OLS) test with a stopwatch, asking the participants to stand unassisted on one leg with eyes open, timed from the other foot leaves the ground till when the foot touches the ground again [26]. A higher score refers to a better balance. The research staff was present to protect the participants from falling during the process.

#### Cognitive function

The Korean version of the Montreal cognitive assessment (K-MoCA) was used to assess cognitive function screening tools with scores ranging from 0 to 30 [27]. The K-MoCA consisted of eight cognitive domains: visuospatial and executive function (5 points), naming (3 points), memory (5 points), attention (6 points), abstraction (2 points), language (3 points), and orientation (6 points) with 12 items. The summed score was adjusted by adding 1 point for those with less than 6-year-educational level. When the MoCA was first developed, the single cut-off score (23 points) was suggested to assess the level of cognitive degradation [28]. Our study included the participants with cognitive impairment when their K-MoCA score was less than 22 points. The reliability and concurrent validity of the K-MoCA have been reported in the Korean population [27].

#### Quality of life

The Korean version of the 12-item Short-Form Health Survey (SF-12) [29] was used to assess the quality of life with eight dimensions. The scores of physical component summary (PCS) and mental component summary (MCS) of QOL were calculated by the standard scoring system and analyzed as separate outcomes as suggested by the developer [29]. The scores ranged from 0 to 100 and higher scores indicated a better quality of life [30]. This scale was validated in the Korean general population with a reliability of 0.88 [29].

### Statistical analysis

**Fig. 1:**
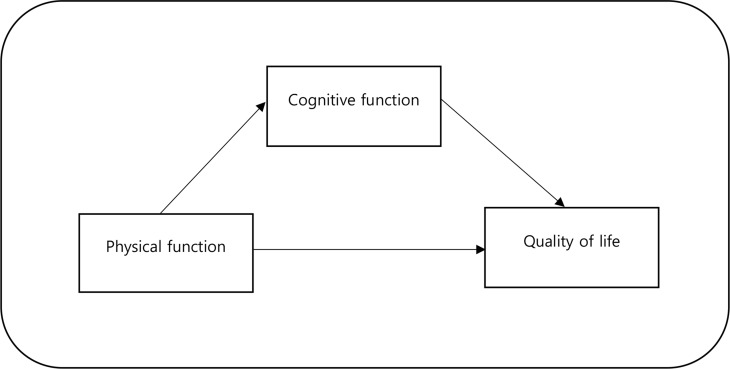
Conceptual framework of physical function as a predictor of quality of life, mediated by cognitive function

The data were analyzed using IBM SPSS Statistics (Version 26.0) with the consideration of a two-tailed *p* < 0.05 to be statistically significant. Descriptive statistics were used for demographic characteristics and study variables with means, standard deviation, and frequency distributions. Pearson’s correlation coefficient analysis was conducted to assess the relationship between the indicators of physical function (Grip strength, TUG, OLS), cognitive function (K-MoCA), and the physical and metal components of QOL (PCS and MCS respectively). Hierarchical multiple linear regression analysis was conducted to enter demographic characteristics (age, sex, and education level) first, followed by physical functions in step 2, and cognitive function in step 3. The variance inflation factor (VIF) values < 10 were considered to be non-collinear [31]. Finally, the mediating effect of cognitive function on the relationship between physical function and QOL was performed using the PROCESS macro for SPSS developed by Hayes [32] with model 4 (http://www.afhayes.com). The number of bootstrap samples was chosen to be 5000, under the bias-corrected 95% confidence interval (CI). The mediation analysis by PROCESS macro was based on regression-based path analysis. We proceeded using the conceptual framework of physical function as a predictor of QOL, mediated by cognitive function (Fig. 1).

## Results

### Characteristics of older adults with MCI

Demographic and clinical characteristics of participants and group differences are shown in Table 1. Of the 79 study subjects, 18 (22.8%) were males, and 61 (77.2%) were females, with a mean age of 77.46 ± 7.03 years (52 ~ 88 years). Thirty-seven participants (46.8%) were widowed, and 23 (29.1%) had high school graduation. Most participants (n = 69; 87.3%) were unemployed, and self-reported economic status was under average (n = 63; 79.7%). Most participants perceived their ADL level as independent (n = 69; 87.3%). Among the history of chronic illness, 45 (60.8%) participants had hypertension, 33 (41.8%) with osteoarthritis, and 23 (29.1%) with diabetes mellitus.

**Table 1:** Demographic characteristics of the participants (*N* = 79)

Characteristics	Categories	n (%)	M ± SD
Gender	Male	18 (22.8)	
	Female	61 (77.2)	
Age (year)	< 60	2 (2.5)	77.46 ± 7.03
	60 ~ 69	7 (8.9)	(range: 52 ~ 88)
	70 ~ 79	37 (46.8)	
	≥ 80	33 (41.8)	
Marital status	Married	28 (35.4)	
	Singled	9 (11.4)	
	Widowed	37 (46.8)	
	Divorced	5 (6.4)	
Education level	None	21 (26.6)	
	Elementary school	21 (26.6)	
	Middle school	10 (12.7)	
	High school	23 (29.1)	
	College or above	4 (5.1)	
Employed	Yes	10 (12.7)	
	No	69 (87.3)	
Economic status	below average	63 (79.7)	
	Average	13 (16.5)	
	above average	3 (3.8)	
Activity of daily living	Independent	69 (87.3)	
	Require assistance	10 (12.7)	
Comorbidity	Hypertension	45 (60.8)	
	Cardiovascular disease	7 (8.9)	
	Diabetes mellitus	23 (29.1)	
	Peripheral vascular disease	3 (3.8)	
	Cerebrovascular disease	11 (13.9)	
	Osteoarthritis	33 (41.8)	
	Mental illness	5 (6.3)	
	Others	27 (34.2)	

### Descriptive data of physical function, cognitive function, and quality of life

The mean physical function scores in participants were 20.53 (SD = 5.72) for grip strength, 11.98 (SD = 5.61) for mobility (TUG), and 4.04 (SD = 4.28) for balance (OLS). The mean cognitive function score was 16.39 (SD = 6.50), ranging from 1 to 29 on a 1–30 scale for the K-MoCA. The mean QOL scores were 44.24 (SD = 9.70) for QOL-PCS and 49.48 (SD = 12.29) for QOL-MCS on a 0-100 sum range.

### Correlations between physical function, cognitive function, and quality of life

Correlations among physical function (grip strength, mobility, and balance), cognitive function, and physical and mental components of QOL (PCS and MCS) were shown in Table 2. Grip strength was positively associated with PCS (r = 0.28, *p* < 0.05). mobility (lower score of TUG represents better motility) was negatively associated with cognition (r=-0.23, *p* < 0.05), PCS (r=-0.32, *p* < 0.01), and MCS (r=-0.42, *p* < 0.01). Balance (OLS) was positively correlated with K-MoCA (r = 0.33, *p* < 0.01).

**Table 2:** Correlation between physical function, cognitive function and quality of life

Variables	Mean (SD)	Score range	Physical function			Cognitive function (K-MoCA)	QOL- PCS
			Grip strength	Mobility (TUG)	Balance (OLS)		
Physical function							
Grip strength	20.53 (5.72)	8.75 ~ 36.25	1				
Mobility (TUG)	11.98 (5.61)	4.95 ~ 37.78	− 0.28^*^	1			
Balance (OLS)	4.04 (4.28)	0 ~ 19.66	0.26^*^	− 0.18	1		
Cognitive function^¶^	16.39 (6.50)	1 ~ 29	0.21	− 0.23^*^	0.33^**^	1	
QOL-PCS^†^	44.24 (9.70)	21.66 ~ 61.25	0.28^*^	− 0.32^**^	0.16	− 0.13	1
QOL-MCS^†^	49.48 (12.29)	16.03 ~ 66.57	0.16	− 0.42^**^	0.09	0.18	0.20

^*^p < 0.05, ^**^p < 0.01;

TUG = Timed up and go, OLS = One leg standing, K-MoCA = Korean version of Montreal cognitive assessment, QOL-PCS = Physical component summary of the quality of life, QOL-MCS = Mental component summary of the quality of life

^¶^Score scale 0–30 (best score 30)

^†^Score scale 0-100 (best score 100, sum of QOL-PCS and QOL-MCS)

### Effects of physical and cognitive function on quality of life

To confirm the direct effect of physical function (Grip strength, TUG, OLS) and cognitive function (K-MoCA) on QOL (PCS and MCS) in older adults with MCI, hierarchical multiple regression analysis was performed on each PCS and MCS of QOL after controlling for demographic characteristics. The results are presented in Tables 3 and 4.

**Table 3:** Physical and cognitive functions to explain the physical component of QOL

Independent variables	PCS					
	Step 1		Step 2		Step 3	
	*β*	*p*	*β*	*p*	*β*	*p*
Age	0.05	0.650	0.11	0.331	0.15	0.146
Gender	0.11	0.330	0.07	0.580	0.02	0.851
Education level	0.08	0.448	0.07	0.595	0.00	0.952
Physical function Grip strength			0.13	0.357	0.23	0.101
Mobility (TUG)			− 0.27	0.024	− 0.31	0.007
Balance (OLS)			0.09	0.434	0.16	0.145
Cognitive function (K-MoCA)					0.34	0.004
F	0.53 (*p* = 0.658)		2.25 (*p* = 0.047)		3.37 (*p* = 0.004)	
R^2^	0.02		0.15		0.25	
Adj R^2^	0.02		0.08		0.17	
△R^2^	0.02		0.13		0.09	

TUG = Timed up and go, OLS = One leg standing, K-MoCA = Korean version of Montreal cognitive assessment, QOL = quality of life

**Table 4:** Physical and cognitive functions to explain the mental component of QOL

Independent variables	MCS					
	Step 1		Step 2		Step 3	
	*β*	*p*	*β*	*p*	*β*	*p*
Age	0.05	0.655	0.11	0.249	0.11	0.256
Gender	0.25	0.028	0.34	0.007	0.34	0.008
Education level	0.13	0.227	0.18	0.067	0.18	0.072
Physical function Grip strength			0.24	0.073	0.24	0.083
Mobility (TUG)			− 0.36	0.002	− 0.36	0.002
Balance (OLS)			0.05	0.607	0.05	0.624
Cognitive function (MoCA)					0.03	0.976
F	2.21 (*p* = 0.094)		5.12 (*p* < 0.001)		4.33 (*p* < 0.001)	
R^2^	0.08		0.29		0.29	
Adj R^2^	0.04		0.24		0.23	
△R^2^			0.21		0.00	

TUG = Timed up and go, OLS = One leg standing, K-MoCA = Korean version of Montreal cognitive assessment, QOL = quality of life

#### Factors to explain PCS

As shown in Table 3, when the independent variables of age, sex, and education level were entered, the regression model was not statistically significant (F = 0.53, *p* = 0.658). In step 2, the demographic characteristics and the indicators of physical function (Grip strength, TUG, OLS) together explained 15% of variables in PCS (F = 2.25, *p* = 0.047), and mobility was the significant predictor after controlling for demographic characteristics. After adding cognitive function (K-MoCA) in step 3, the regression model was significant (F = 3.37, *p* = 0.004), explaining 25% of the variance in PCS. Cognitive function (*β* = 0.34, *p* = 0.004) and mobility (*β*=-0.31, *p* = 0.007) were significant predictors of PCS after controlling for demographic characteristics.

#### Factors to explain MCS

As shown in Table 4, the regression model with independent variables of age, sex, and education level was not statistically significant (F = 2.21, *p* = 0.094). In step 2, the demographic characteristics and the indicators of physical function (Grip strength, TUG, OLS) together explained 29% of variables in MCS (F = 5.12, *p <* 0.001), and mobility was also the significant predictor after controlling for demographic characteristics. After adding cognitive function (K-MoCA) in step 3, the regression model was significant (F = 4.33, *p <* 0.001), explaining 29% of the variance in MCS. Cognitive function was not a significant factor (*β* = 0.03, *p* = 0.976), while mobility (*β*=-0.36, *p* = 0.002) was a significant predictor of MCS after controlling for demographic characteristics.

#### Mediating effects of cognitive function between physical function and quality of life

**Fig. 2:**
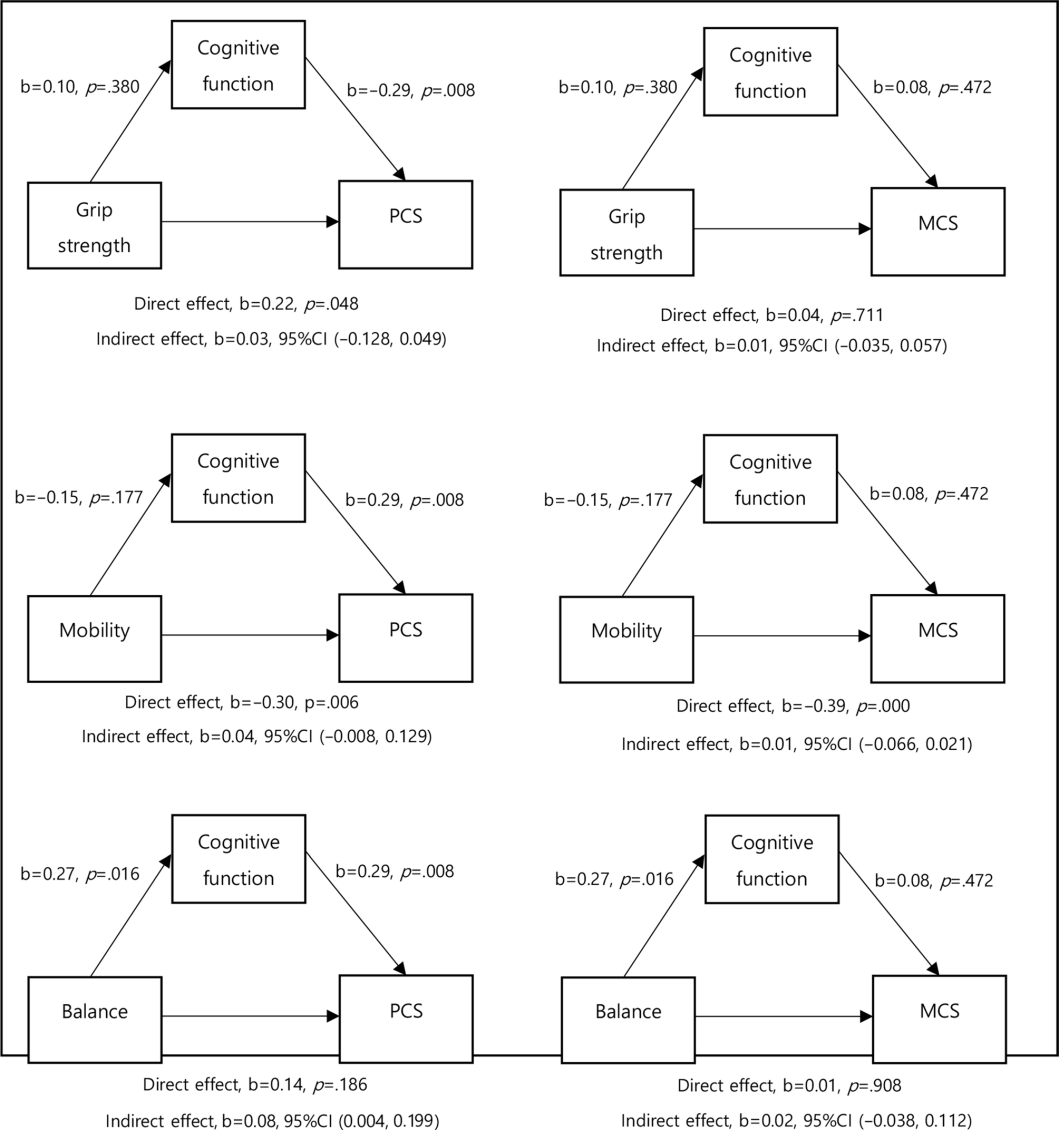
Effects of the indicators of physical function (Grip strength, mobility, balance) on the physical and mental components of quality of life (PCS and MCS) as cognitive function as a mediator *p < 0.05, **p < 0.001;

The indirect effects of physical function (grip strength, mobility, and balance) on PCS and MCS with cognitive function as mediators were examined (Fig. 2). Among the indicators of physical function, balance has a significant indirect effect through cognitive function on PCS (b = 0.08, 95% CI 0.004,0.199).

## Discussion

This study aimed to verify the mediating effect of cognitive function in the relationship between physical function (grip strength, mobility, and balance) and physical and mental components of QOL in older adults with MCI.

Among the indicators of physical function, mobility was the most significant factor in predicting PCS and MCS in older adults with MCI after controlling for age, sex, and education level. Previous studies support this finding, confirming the relationship between mobility and quality of life among older adults [33, 34]. The Timed Up and Go (TUG) test is simple and widely used in clinical practice as a reliable measurement to quantify mobility performance in persons with cognitive impairment [35, 36]. The slower performance of TUG (less mobility) was associated with lower cognitive function in individuals with Parkinson’s disease; both were the most significant predictors of QOL in individuals with Parkinson’s disease [35]. In addition, studies focusing on MCI with QOL found a significant impact of cognitive impairment status on the autonomy facet of QOL [6]. Given that impaired mobility contributes to loss of functional independence leading to reduced QOL, mobility could be related to autonomy [6, 34]. Older people with cognitive impairment are at high risk for falls due to mobility impairment, and is associated with greater risk for institutionalization and increased mortality [34]. Impaired mobility was a critical issue for older adults. Consequently, mobility would be an important predictor of changes in QOL in later life [34].

While balance was positively correlated with cognitive function in our study, there was no significant direct effect of balance on both PCS and MCS in older adults with MCI. While balance impairments are common in individuals with cognitive impairment [37], improving balance can play an essential role in providing strategies to prevent falls which is one of the crucial factors for QOL in older adults with MCI [38]. In a previous study, however, a 12-month program of a variety of exercises significantly improved balance in older adults with MCI, but no significant effect on QOL [39]. As previous studies have found that improvements in postural control are associated with cognitive training, the role of cognitive function needs to be explored to understand the underlying mechanism of balance and QOL in older adults with cognitive impairment [37, 40].

Our study explored the relationship between physical function and QOL with cognitive function as a mediator. While there was no significant direct effect of balance on QOL, our finding indicated the significant indirect effect of balance on PCS through cognitive function. In the context of anatomical and functional changes in MCI, the balance is seen as a result of the complex integration and coordination of multiple basic systems covering sensory/perceptual processes, cognitive influences, and motor processes [41]. The balance training for 12 weeks improved memory and spatial cognition in healthy adults [42], and 2-week balance training improved postural control, which was associated with improved intracortical inhibition [43]. It has been speculated that when balance training sends signals to specific areas of the brain, it may affect cognitive functions and that increased stimulation of the vestibular system during balance exercise may be a mediator between physical movement and cognitive function [44]. Therefore, enhancing the balance of physical function can improve the QOL among older adults with MCI through improving cognitive function, which supports the findings of our study. However, this assumption was supported only by the physical component of QOL, not by the mental component (MCS). This finding could be explained by the fact that the interaction between physical function (i.e., balance) and cognitive function would be more closely associated with the physical component of QOL compared to the MCS. In addition, the limited variance in cognitive function of the study participants with MCI could contribute to non-significant findings. Based on the results, the underlying mechanism of physical and cognitive function to the mental component of QOL is warranted for further investigation.

Grip strength is a useful index of aging that is easy to perform as a comprehensive indicator of whole-body muscle strength [45]. Previous studies suggested that grip strength may be used as an indicator of overall strength, function, and QOL [46, 47], but we found no significant direct effects between grip strength and both PCS and MCS in older adults with MCI. The relationship between grip strength and cognitive decline was age-dependent, which could be stronger in more advanced stages of cognitive decline rather than in the early cognitive decline [24]. The inclusion criteria for the present study was mild cognitive impairment with an average K-MoCA score of 16.39, which may be the reason for the non-significant association between grip strength and QOL found in the present study.

There were limitations in our study. First, we found no significant indirect effect of balance on the mental component of QOL through cognitive function. It could be due to the limited variance in cognitive function since this secondary analysis was conducted with older adults with cognitive impairment. It would be necessary to explore the mediating effects of cognitive function in the relationship between physical function and quality of life among older adults with various levels of cognitive function, including healthy population. In addition, our study assessed cognitive function using a single measurement of MoCA. Future studies are warranted with systematically assessing multi-dimensions of cognitive function and a longitudinal design to add evidence to the present results.

## Conclusion

Physical and cognitive functions are important factors that can predict QOL of older adults with cognitive impairment. Among the indicators of physical function, mobility was the significant predictor of the physical and mental components of QOL. Cognitive function partially mediated the relationship between OLS of physical function and PCS of QOL in older adults with MCI. Health promoting strategies should focus on mobility and balance in association with cognitive function to delay or prevent cognitive decline and to promote QOL in older adults with MCI.

## Data Availability

The datasets used and/or analyzed during the current study are available from the corresponding author on reasonable request.
