# Correction to: Physical and cognitive function to explain the quality of life among older adults with cognitive impairment: exploring cognitive function as a mediator

**DOI:** 10.1186/s40359-023-01108-3

**Published:** 2023-03-07

**Authors:** Rhayun Song, Xing Fan, Jisu Seo

**Affiliations:** College of Nursing, Chungnam National University, Munhwa-ro 266, Jung-gu, Daejeon, Korea

**Correction to: BMC Psychology (2023) 11:51**

10.1186/s40359-023-01087-5

Following publication of the original article [1], the authors flagged that the first and family name of the first author, Rhayun Song, had been erroneously swapped. The name has since been corrected in the published article.

